# ADDRESSING THE MENTAL HEALTH NEEDS OF HOMEBOUND OLDER ADULTS IN AGING SERVICE SETTINGS

**DOI:** 10.1093/geroni/igac059.386

**Published:** 2022-12-20

**Authors:** Namkee Choi

**Affiliations:** University of Texas at Austin, Austin, Texas, United States

## Abstract

Despite significant and urgent mental health needs among low-income homebound older adults, the existing mental health service systems’ reach for these older adults is almost nonexistent. Given the current and projected geriatric mental health workforce shortages, innovative approaches are needed to better serve these underserved older adults. This presentation will focus on a series of randomized clinical trials that tested acceptable and feasible mental health service delivery models for homebound older adults who are served by aging service network agencies that provide hot meals and case management. Findings from the real-world effectiveness trials of tele-delivered behavioral activation treatment for depression and loneliness by bachelor’s-level lay counselors who are embedded in aging service agencies will be shared. Ways to promote a wider adoption of scalable and sustainable mental health service delivery models for homebound older adults will be discussed.

